# Very early virological failure and drug resistance mutations in a woman on antiretroviral therapy in Eastern Cape, South Africa: a case report

**DOI:** 10.1186/s13256-015-0557-0

**Published:** 2015-05-07

**Authors:** Olufunso Oladipo Sogbanmu, Oladele Vincent Adeniyi, Yusimi Ordaz Fuentes, Daniel Ter Goon

**Affiliations:** 1 Department of Family Medicine, Division of HIV Care, Cecilia Makiwane Hospital, East London Hospital Complex, East London, Eastern Cape Province South Africa; 2 School of Health Sciences, University of Fort Hare, East London, South Africa

**Keywords:** Genotypic, Guidelines, HIV, Primary HIV resistance, Transmitted drug-resistant virus

## Abstract

**Introduction:**

Rapid scale-up of antiretroviral therapy rollout in Sub-Saharan African countries faces the challenge of virological failure. This could be the consequence of transmitted drug-resistant human immunodeficiency virus strains at the population level. While a pre-antiretroviral therapy genotypic test has been a major component of the human immunodeficiency virus management programme in developed nations, it is yet to be incorporated into the antiretroviral therapy programme in resource-poor countries.

**Case presentation:**

A 32-year-old Black African woman was seen for her six-month routine review. Her viral load after initiation of fixed drug combination of tenofovir, emtricitabine and efavirenz was 31,397 RNA copies/mL. Adherence was assessed to be good based on pharmacy pick-up dates, on-time clinic appointment records, medical file review, self-reporting and treatment supporter’s report. Her viral load was repeated after another two months of close monitoring; the result showed viral load of 31,159 RNA copies/mL. She was assessed as virological failure to her first-line antiretrovirals and commenced on second-line antiretrovirals: zidovudine/lamivudine/Aluvia^®^ (lopinavir and ritonavir). A human immunodeficiency virus drug genotypic testing showed she was only susceptible to zidovudine and protease inhibitors. At third month on the new regimen, her viral load was suppressed.

**Conclusions:**

This case report demonstrates the possibility of a silent epidemic within the human immunodeficiency virus pandemic in resource-poor settings like Eastern Cape, South Africa. We described a case of early virological failure in a highly motivated young woman. Although, a pre-antiretroviral therapy genotypic test is yet to be incorporated into a human immunodeficiency virus programme in resource-poor countries, the need for it might become evident as the programme expands. Close monitoring of the viral load of patients according to national guidelines will enable early detection of a failing regimen and prompt intervention can be instituted to prevent morbidity and mortality. There is an urgent need to strengthen the human immunodeficiency virus programme in resource-poor countries to prevent the emergence of an epidemic of transmitted drug-resistant human immunodeficiency virus strains within the existing human immunodeficiency virus pandemic.

## Introduction

According to the 2013 Global Report of the Joint United Nations Programme on HIV and AIDS (UNAIDS), 35.3 million people were living with human immunodeficiency virus (HIV) by the end of 2012 and 70.8% of the global burden reside in Sub-Saharan Africa. South Africa has the highest burden of the HIV epidemic (6.4 million people living with HIV/acquired immunodeficiency syndrome) [1]. The global response to the epidemic has seen more than 60% of eligible individuals already initiated on antiretroviral therapy (ART) using World Health Organization (WHO) criteria of 2010 (CD4 count ≤350 cells/mL) and approximately 34% using WHO 2013 criteria (CD4 count ≤500 cells/mL) [2].

The impact of the scale-up of ART includes 4.2 million deaths averted, 800,000 child infections averted with the Prevention of Mother-to-Child Transmission of HIV programme and a decrease in the incidence of HIV infections in 2012 [2]. The probable challenges of rapid scale-up of ART include loss to follow up, virological failures and drug resistance (acquired and transmitted). These have not been fully investigated in resource-poor settings; however, their possibility has been documented [3]. While acquired resistance has received attention in the national programmes of most resource-poor countries of Sub-Saharan Africa [4], transmitted resistance has not received similar attention.

This is the first case report to the best of our knowledge to describe a patient who experienced a primary virological failure possibly secondary to transmitted drug-resistant HIV (TDRHIV) strain in Eastern Cape, South Africa.

## Case presentation

Six months ago, a 32-year-old Black African woman was seen for her routine review. She was asymptomatic. Approximately 13 to 14 months earlier, she was referred to our HIV Unit because of a low CD4 count for ART initiation. She was antiretroviral (ARV)-naïve with WHO clinical stage 3 (oral candidiasis) and a baseline CD4 count of 14 cells/mL. Reflex laboratory serum Cryptococcal Latex Antigen Test serology was negative. The results of other baseline investigations were within normal limits. Her symptom screen for tuberculosis was negative. Alcohol and substance use were excluded. There was no history of depression or any comorbid chronic illnesses. She disclosed her status to her partner. She was nulliparous.

Her medications during preparation for ARVs included co-trimoxazole, multivitamins and oral nystatin. She was commenced on a fixed dose combination of tenofovir, emtricitabine and efavirenz daily (according to the South African National Department of Health Guideline, 2013) [4]. She was followed up at 2, 4 and 8 weeks for immune reconstitution inflammatory syndrome and adverse effects. At each visit, adherence was reviewed and further counselling was provided. Isoniazid prophylaxis was initiated; her estimated glomerular filtration rate was normal at three months (glomerular filtration rate >60 mL/minute/1.73m^2^).

She was again reviewed at six months in 2014: viral load (VL) of 31,397 ribonucleic acid (RNA) copies/mL; a log value of 4 was obtained. Poor adherence was considered despite her and the treatment supporter’s account of good adherence. A thorough evaluation of the medical files for clinic attendance, pharmacy records for pick-up of medications and pill count charts was conducted. She was deemed to have a relatively good adherence. There were no drug–drug interactions; no herbal or alternative therapies were used during the period of treatment. She was not treated for diarrhoea or vomiting during the preceding 6 months. Further questions about her new sexual partner and HIV status of previous sexual partners did not yield any new information and she had not been sexually active since diagnosis of HIV. Adherence was consolidated for another two months with intense monthly review. Her VL at eight month remained 31,159 RNA copies/mL in spite of excellent adherence. We confirmed virological failure. An assessment of a probable primary virological failure from TDRHIV was considered. A decision was made to switch to a second-line regimen of zidovudine (AZT)/lamivudine/lopinavir and ritonavir at standard doses.

After analysing the case we decided to do an HIV drug genotypic test outside the National guideline to understand the pattern of resistant mutation in our patient.

The genotypic test result showed susceptibility to protease inhibitors and AZT (Table 1).

**Table 1:** **Mutation score of patient**

**RT**	**3TC**	**ABC**	**AZT**	**D4T**	**DDI**	**FTC**	**TDF**	**EFV**	**ETR**	**NVP**	**RPV**
K65R	30	45	−15	45	60	30	60	−	−	−	−
M184I	60	15	−10	−10	10	60	−10	−	−	−	−
K103S	−	−	−	−	−	−	−	45	0	60	0
V106M	−	−	−	−	−	−	−	60	0	60	0
M230L	−	−	−	−	−	−	−	45	30	60	45
K653+M184I	−	−	−	10	−	−	10	−	−	−	−

*Abbreviations*: *3TC* lamivudine, *ABC* abacavir, *AZT* zidovudine, *D4T* stavudine, *DDI* didanosine, *EFV* efavirenz, *ETR* etravirine, *FTC* emtricitabine, *NVP* nevirapine, *RPV* rilpivirine, *RT* reverse transcriptase, *TDF* tenofovir.

The HIV subtype isolated in the genotypic testing was subtype C. The K65R mutation was noted as part of the nucleoside/nucleotide reverse-transcriptase inhibitor mutation; however, AZT remains active.

She was reviewed monthly and a blood sample for VL was taken at third month of commencing second-line ARVs. The result confirmed viral suppression (VL=25 RNA copies/mL). She is presently doing well.

**Figure 1:**
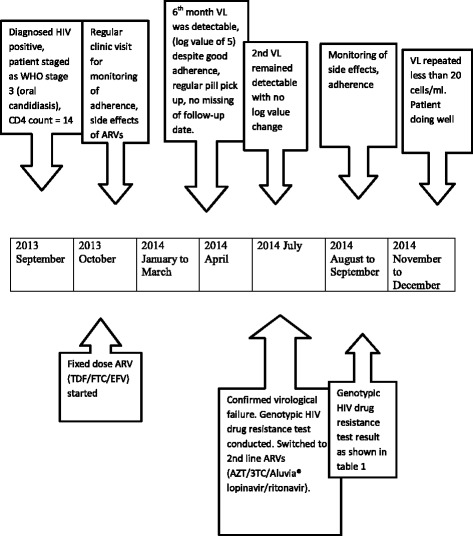
Timeline of important clinical events during management of patient. Abbreviations: 3TC, lamivudine; ARV, antiretroviral; AZT, zidovudine; EFV, efavirenz; FTC, emtricitabine; HIV, human immunodeficiency virus; RNA, ribonucleic acid; TDF, tenofovir; VL, viral load; WHO, World Health Organization. Aluvia® is lopinavir and ritonavir.

The timeline of the case is shown in Figure 1.

## Discussion

The case report suggests the possibility of yet another epidemic that may be emanating from the HIV pandemic; awareness of its existence is a reality in resource-poor countries. Whether our case represents a part of the rising prevalence of TDRHIV remains uncertain. A pre-ART genotypic test is a prerequisite for initiation of treatment in resource-rich nations [5], whereas a genotypic test is reserved for second-line regimen failures in resource-constrained settings like ours [4,6,7]. The difference between the two models of HIV care could result in a minimum of ≥eight months’ delay in initiating effective ART in patients with TDRHIV which was the case in this patient.

The two VLs taken according to the South African ART guideline [4] confirmed virological failure. What remains applicable in our practice setting is the role of VL monitoring, which was adhered to strictly in our patient. Hence, VL monitoring may remain the only tool to determine early ARV failure in the resource-poor setting where we practice. However, it is worth noting that TDRHIV is one of the documented causes of virological failure on a first-line regimen [8] which is a possibility in this patient. Safe sexual practice should be advocated in all patients to prevent the spread of TDRHIV strains at a community level [9,10]. Of importance in our patient is her being ARV naïve with no past obstetrics history.

Similar to a meta-analysis conducted by Gupta *et al*., our patient has acquired many mutations (Table 1) [11]. However, we found that she remained susceptible to AZT as mutations found in combination with K65R in our case enhance susceptibility to AZT (M184V as well as K65R) [12]. Therefore, we expect that AZT would remain active against such viruses. The latest VL of 25 RNA copies/mL from her confirmed an effective ART after an initial delay of 8 months.

### Limitations of current practice

It remains a challenge that a pre-ART genotypic resistance testing could not be undertaken in patients with HIV infection in a resource-constrained setting as this would have provided information on the baseline ARV drug resistance. Also, the inability to conduct ARV drug levels in the partner of the patient should be noted.

## Conclusions

Although, a pre-ART genotypic test is yet to be incorporated into an HIV programme in resource-poor countries, the need for its incorporation into clinical care may become more evident as the prevalence of TDR rises. Meanwhile, close monitoring of VL of patients according to the national guidelines is very important. This will enable early detection of a failing regimen and prompt intervention can be instituted to prevent accumulation of resistant strains, virological failure, morbidity and mortality. There is an urgent need to strengthen the HIV programme in resource-poor settings to prevent the emergence of a silent epidemic of TDRHIV within the HIV pandemic.

## Consent

Written informed consent was obtained from the patient for publication of this case report and any accompanying images. A copy of the written consent is available for review by the Editor-in-Chief of this journal.

## Abbreviations

ART: Antiretroviral therapy
ARV: Antiretroviral
AZT: Zidovudine
HIV: Human immunodeficiency virus
RNA: Ribonucleic acid
TDRHIV: Transmitted drug-resistant HIV
UNAIDS: Joint United Nations Programme on HIV and AIDS
VL: Viral load
WHO: World Health Organization
